# Azido­{1-[(2-diethyl­amino­ethyl­imino)meth­yl]naphthalato}copper(II). Erratum

**DOI:** 10.1107/S2056989015022264

**Published:** 2016-08-31

**Authors:** Xiao-Yang Qiu

**Affiliations:** a Department of Chemistry, Fuyang Normal College, Fuyang Anhui 236041, People’s Republic of China

## Abstract

In the paper by Qiu [*Acta Cryst.* (2006), E**62**, m849–m850], the metal atom was reported incorrectly.

## Abstract 2

Erratum to *Acta Cryst.* (2006), E**62**, m849–m850.

The author of Qiu (2006) ▸ has indicated that the metal atom in the reported complex was assigned incorrectly. The paper is therefore withdrawn from the published literature.
